# Suspected common bile duct stones: reduction of unnecessary ERCP by pre-procedural imaging and timing of ERCP

**DOI:** 10.1007/s00464-022-09615-x

**Published:** 2022-09-26

**Authors:** Christina J. Sperna Weiland, Evelien C. Verschoor, Alexander C. Poen, Xavier J. M. N. Smeets, Niels G. Venneman, Abha Bhalla, Ben J. M. Witteman, Hester C. Timmerhuis, Devica S. Umans, Jeanin E. van Hooft, Marco J. Bruno, P. Fockens, Robert C. Verdonk, Joost P. H. Drenth, Erwin J. M. van Geenen, for the Dutch Pancreatitis Study Group

**Affiliations:** 1 Department of Gastroenterology and Hepatology, Radboudumc, Postbus 9101, 6500 HB Nijmegen, The Netherlands; 2 Department of Research and Development, St. Antonius ziekenhuis, Nieuwegein, The Netherlands; 3 Department of Gastroenterology and Hepatology, Isala Clinics, Zwolle, The Netherlands; 4 Department of Gastroenterology and Hepatology, Jeroen Bosch Ziekenhuis, Den Bosch, The Netherlands; 5 Department of Gastroenterology and Hepatology, Medisch Spectrum Twente, Enschede, The Netherlands; 6 Department of Gastroenterology and Hepatology, Hagaziekenhuis, The Hague, The Netherlands; 7 Department of Gastroenterology and Hepatology, Gelderse Vallei Hospital, Ede, The Netherlands; 8 Department of Surgery, St. Antonius ziekenhuis, Nieuwegein, The Netherlands; 9 Department of Gastroenterology and Hepatology, Amsterdam Gastroenterology & Metabolism, Amsterdam UMC, Amsterdam, The Netherlands; 10 Department of Gastroenterology and Hepatology, Leiden University Medical Center, Leiden, The Netherlands; 11 Department of Gastroenterology and Hepatology, Erasmus Medical Center, Rotterdam, The Netherlands; 12 Department of Gastroenterology and Hepatology, St. Antonius ziekenhuis, Nieuwegein, The Netherlands

**Keywords:** Choledocholithiasis, Gallstones, Cholangiopancreatography, Endoscopic retrograde, Cholangiopancreatography magnetic resonance

## Abstract

**Background:**

Endoscopic retrograde cholangiopancreatography (ERCP) is the procedure of choice to remove sludge/stones from the common bile duct (CBD). In a small but clinically important proportion of patients with suspected choledocholithiasis ERCP is negative. This is undesirable because of ERCP associated morbidity. We aimed to map the diagnostic pathway leading up to ERCP and evaluate ERCP outcome.

**Methods:**

We established a prospective multicenter cohort of patients with suspected CBD stones. We assessed the determinants that were associated with CBD sludge or stone detection upon ERCP.

**Results:**

We established a cohort of 707 patients with suspected CBD sludge or stones (62% female, median age 59 years). ERCP was negative for CBD sludge or stones in 155 patients (22%). Patients with positive ERCPs frequently had pre-procedural endoscopic ultrasonography (EUS) or magnetic resonance cholangiopancreatography (MRCP) imaging (44% vs. 35%; *P* = 0.045). The likelihood of ERCP sludge and stones detection was higher when the time interval between EUS or MRCP and ERCP was less than 2 days (odds ratio 2.35; 95% CI 1.25–4.44; *P* = 0.008; number needed to harm 7.7).

**Conclusions:**

Even in the current era of society guidelines and use of advanced imaging CBD sludge or stones are absent in one out of five ERCPs performed for suspected CBD stones. The proportion of unnecessary ERCPs is lower in case of pre-procedural EUS or MRCP. A shorter time interval between EUS or MRCP increases the yield of ERCP for suspected CBD stones and should, therefore, preferably be performed within 2 days before ERCP.

**Graphical abstract:**

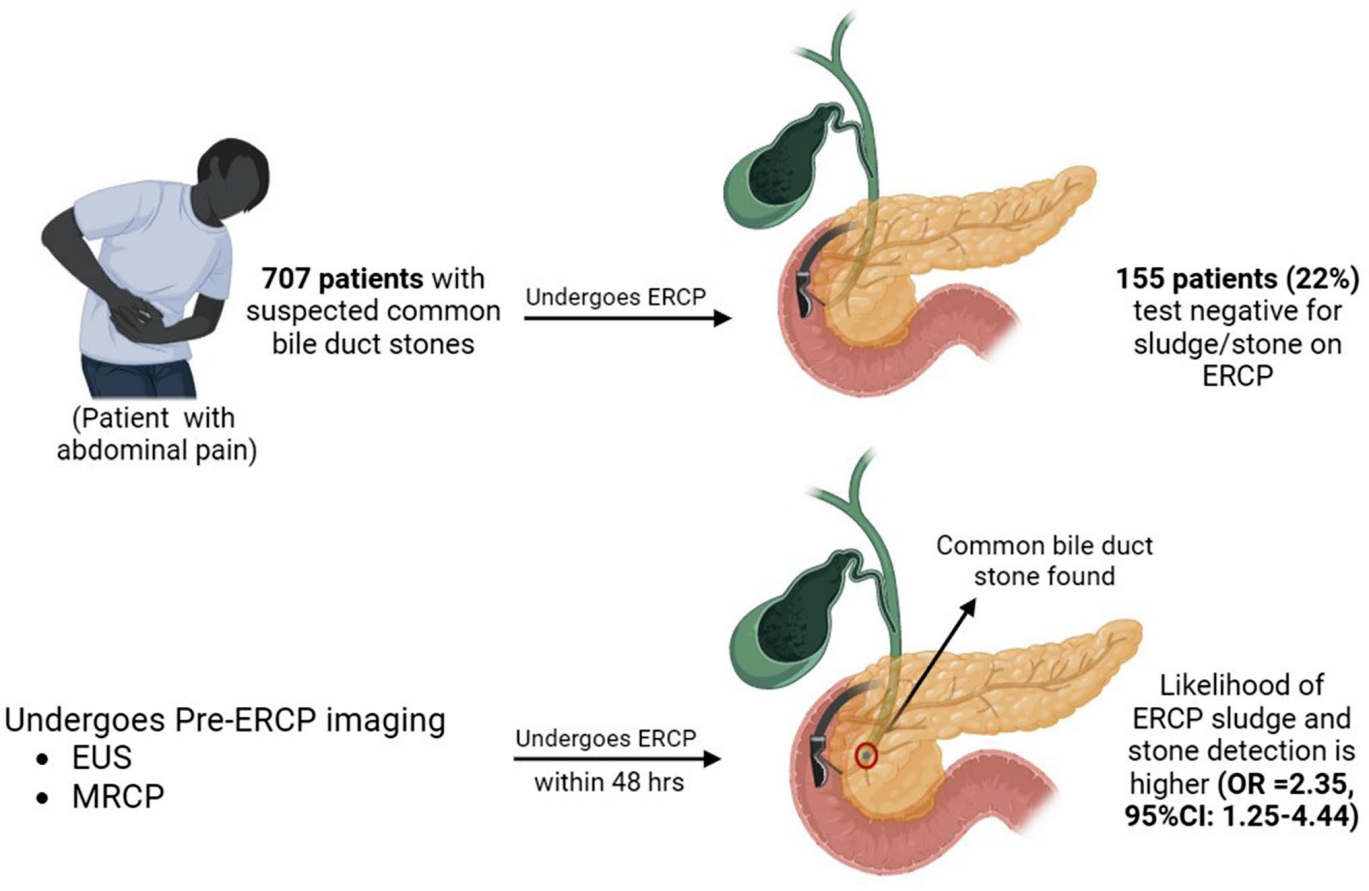

**Supplementary Information:**

The online version contains supplementary material available at 10.1007/s00464-022-09615-x.

Common bile duct (CBD) stones are a commonly occurring gastroenterological condition, with an estimated yearly incidence of 45 per 100,000 in Western populations, increasing with age [1–3]. CBD stones can lead to symptoms and complications, such as abdominal pain, jaundice, infection, and acute pancreatitis [4]. Guidelines recommend removal of CBD stones with endoscopic retrograde cholangiopancreatography (ERCP) [4–6].

Although ERCP is considered the gold standard for the treatment of CBD stones, the diagnostic role of this modality is limited by its invasive nature and safety issues, most notably the risk of post-ERCP pancreatitis (3.5–9.7%), cholangitis (0.5–3.0%), bleeding (0.3–9.6%), perforation (0.08–0.6%), and anesthesia-related adverse events (0.02%) [7, 8]. Therefore it is important to curtail ERCP use to patients with the highest likelihood of CBD sludge or stones. The recommended diagnostic work-up consists of liver biochemistry and abdominal ultrasound (US) or cross-sectional imaging [4, 6]. In order to avoid unnecessary ERCPs, guidelines stratify patients with suspected CBD stones into three risk categories. Patients with a low likelihood (< 10%) of CBD stones are recommended for a prompt cholecystectomy without prior ERCP. Patients with a persistent clinical suspicion of CBD stones, but insufficient evidence of stones on abdominal US or cross-sectional imaging, are stratified with an intermediate likelihood (10–50%) of CBD stones. These patients are recommended to receive further imaging with endoscopic ultrasound (EUS) or magnetic resonance cholangiopancreatography (MRCP), representing less-invasive and lower-risk alternatives for initial evaluation. For patients with a high likelihood (> 50%) it is advised to proceed directly to an ERCP procedure for stone clearance. However, even in patients with a high likelihood of CBD stones established after appropriate diagnostic work-up, 17–57% do not have stones or sludge on ERCP while being exposed to the risk of developing potential harmful complications [9–14].

We aimed to map the diagnostic pathway leading up to ERCP in current clinical practice and evaluate the ERCP outcome in a prospectively collected multicenter cohort of patients with suspected CBD stones.

## Materials and methods

### Study design and setting

We created a prospective cohort of patients with suspected CBD stones from a nationwide multicenter, parallel-group open-label randomized controlled superiority trial [15]. Briefly, this trial investigated whether aggressive periprocedural hydration with lactated Ringer’s solution additional to standard rectal non-steroidal anti-inflammatory drugs (NSAIDs) could prevent post-ERCP pancreatitis in moderate-to-high-risk patients. The study received permission from the Medical Research Ethics Committees United (NL52341.100.15, April 14, 2015). Written informed consent was obtained from all participants. The study included 826 adult patients (18–85 years), from 22 Dutch hospitals, who underwent ERCP between June 2015 and June 2019. This study adheres to the Strengthening the Reporting of Observational studies in Epidemiology (STROBE) guideline.

### Participants

Patients with ongoing acute pancreatitis were not included in the original trial. For the current study, patients were excluded when no ERCP was performed, if suspected CBD stones was not the indication for performing the ERCP, or when the presence of CBD sludge/stones could not be assessed during ERCP due to technical failure. ERCPs were performed in an outpatient and inpatient setting.

### Data collection

During the trial, data were prospectively collected using standardized case record forms. The study coordinator verified these through patient chart review. We collected the following data for each patient: presence of fever (> 38 °C) or/and chills before ERCP, biochemical test results within 1 month prior to ERCP, and results from abdominal imaging (abdominal US, computed tomography (CT), EUS, or MRCP) performed in the 3 months preceding ERCP. In case abdominal US or CT was performed, this was scored as initial imaging according to the guidelines, while performance of EUS or MRCP was considered additional imaging.

### Outcomes and definitions

The primary endpoint was the proportion of ERCPs negative for sludge or stones in the CBD, irrespective of other ERCP findings (e.g., cholangiocarcinoma, papillary stenosis). The ERCP was considered positive upon clear photographic documentation of filling defect on cholangiography or when the endoscopist’s report documented stones or any sludge. ERCP was considered negative in all other cases. Work-up toward ERCP was left at the discretion of the treating clinician. For this reason, we were able to assess the endpoint in a cohort of patients which reflects real-world practice work-up (whole cohort) and a subgroup of patients who have had a work-up as defined by the American Society for Gastrointestinal Endoscopy (ASGE) 2019 guidelines. This subgroup included patients stratified to intermediate likelihood of CBD stones who underwent additional imaging and patients stratified to high likelihood of CBD stones who went straight to ERCP.

Each patient was retrospectively categorized in a low, intermediate, or high likelihood of CBD stones, according to the ASGE guideline for endoscopic management of CBD stones of 2010 and 2019 [4, 5] and the European Society for Gastrointestinal Endoscopy (ESGE) guideline of 2019 [6]. Patients were considered to have a high likelihood for CBD stones according the ASGE 2019 guideline when they met one of the following criteria: CBD stone on abdominal US/cross-sectional imaging, clinical ascending cholangitis, or combination of total bilirubin > 4 mg/dL and dilated CBD on abdominal US/cross-sectional imaging. Intermediate likelihood for CBD stones when they met one of the following criteria: abnormal liver biochemical tests, age > 55 years or dilated CBD on abdominal US/cross-sectional imaging. All other patients were considered as having low likelihood for CBD stones. Clinical ascending cholangitis was defined according to the 2018 Tokyo Guideline [16]. See Appendix Table S1, for details of ASGE and ESGE guideline criteria.

Secondary endpoints included the effects of pre-ERCP imaging on CBD sludge/stone visualization during ERCP, the timing (in days) of pre-ERCP imaging, and the ERCP-related complication rate in relation to presence of CBD stones. We used the Cotton criteria to describe ERCP-related complications (pancreatitis, cholangitis, bleeding, and perforation) [17].

### Statistical analysis

Baseline variables were assessed by mean with standard deviation (SD) or median with interquartile range (IQR). Primary and secondary endpoints were assessed using Mann–Whitney *U* test, Pearson *χ*^2^ test, or Fisher exact test as appropriate. All statistical analyses were conducted using IBM SPSS Statistics for Windows, version 24, with statistical significance set at a two-sided alpha level of 5%.

## Results

### Cohort identification and characteristics

**Fig. 1:**
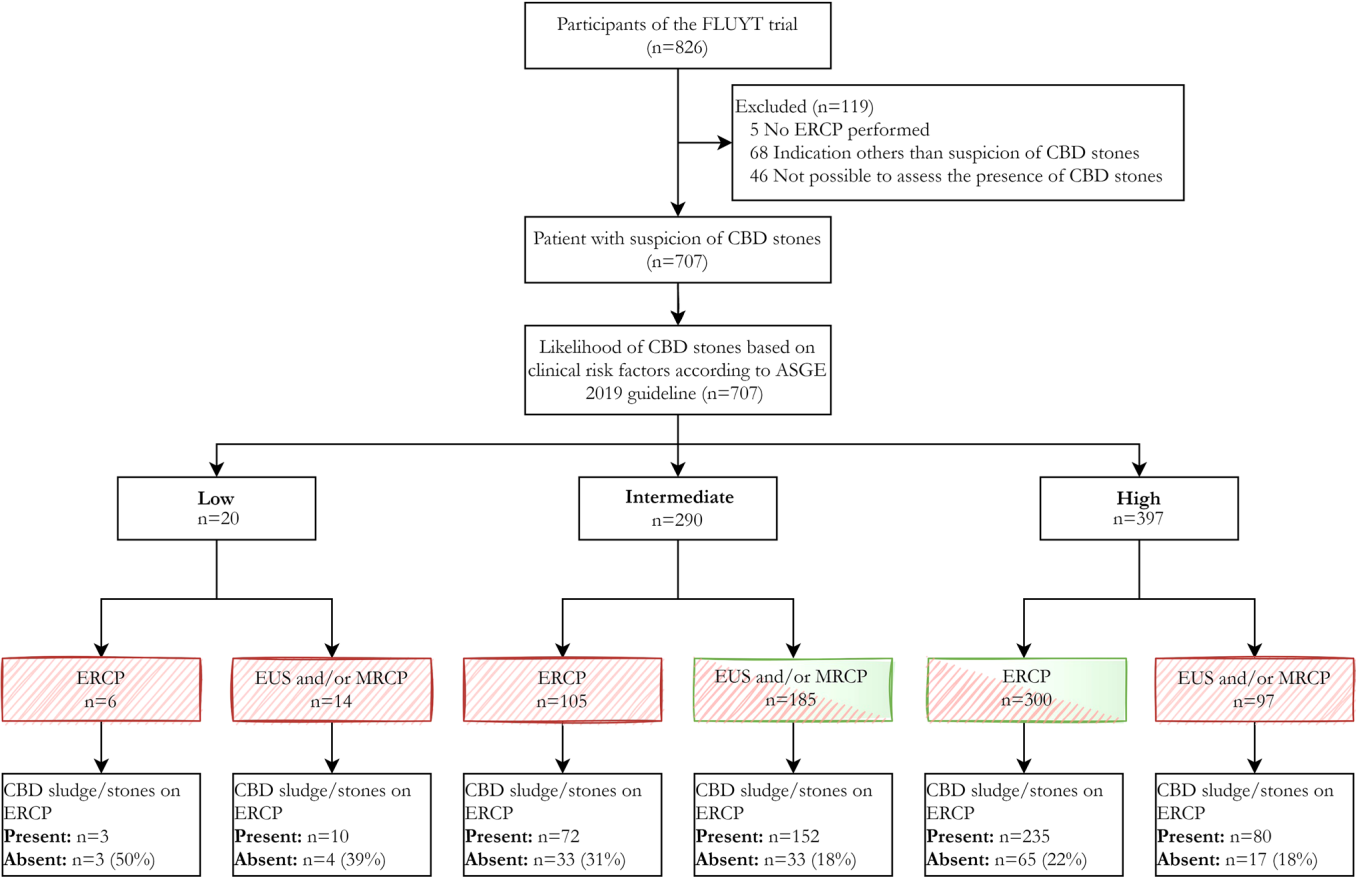
Patient selection and risk stratification of included patients with suspected common bile duct stones. *ASGE* American society for gastrointestinal endoscopy, *CBD* common bile duct, *ERCP* endoscopic retrograde cholangiopancreatography *EUS* endoscopic ultrasonography, *MRCP* magnetic resonance cholangiopancreatography. *Fully striped box* work-up according to real-world practice, *Half striped box* work-up according to ASGE 2019 guideline

A total of 826 patients were enrolled in the original multicenter randomized trial. We excluded 119 patients (Fig. 1), because of an indication for ERCP other than (suspected) stones in the CBD (*n* = 68), procedure failure of ERCP (e.g., failed cannulation, ampulla not reached) (*n* = 46), and no performance of ERCP (*n* = 5). Finally, we included a total of 707 patients in the current study.

The median age of the patients was 59 years, and 436 (62%) patients were female (Table 1). The majority of the patients (92%) had biochemical tests examined prior to ERCP. Abdominal US was performed in 617 (87%) patients and CT in 90 (13%) patients. 338 (48%) patients suffered from acute cholangitis. According to the ASGE 2019 guideline, 20 patients were considered as low likelihood, 290 patients as intermediate likelihood, and 397 patients as high likelihood for choledocholithiasis. 485 patients (69%) received work-up as recommended by the ASGE 2019 guideline. See Appendix Table S2, for risk stratification and results of the ASGE 2010 and ESGE 2019.

**Table 1:** Patient characteristics

	*N* (%)	Median (IQR)
Age (years)		59 (46–71)
Female sex	436 (62%)	
Body mass index		27 (24–30)
Previous cholecystectomy	187 (26%)	
Cholangitis*	338 (48%)	
Biochemical test values	644 (91%)	
Days before ERCP		1 (0–3)
Total bilirubin (umol/L) > ULN	470/632 (74%)	44 (16–84)
ALT (U/L) > ULN	586/635 (92%)	278 (116–446)
AST (U/L) > ULN	538/612 (88%)	141 (68–245)
ALP (U/L) > ULN	520/618 (84%)	218 (137–323)
GGT (U/L) > ULN	599/628 (95%)	430 (214–727)
Abdominal US	617 (87%)	
CBD stone or sludge	191 (31%)	
Dilated CBD	387 (63%)	
Previous cholecystectomy	81	
No previous cholecystectomy	306	
CT-scan	90 (13%)	
CBD stone or sludge	41	
Dilated CBD	65	
Imaging positive for choledocholithiasis prior to ERCP	296 (42%)	
MRCP	71/82 (87%)	
EUS	216/225 (96%)	

*According to Tokyo Guidelines 2018

*AST* Aspartate transaminase, *ALT* Alanine transaminase, *ALP* Alkaline phosphatase, *GGT* Gamma-glutamyltransferase, *US* Ultrasound, *CT* computed tomography, *EUS* endoscopic ultrasonography, *MRCP* magnetic resonance cholangiopancreatography, *ERCP* endoscopic retrograde cholangiopancreatography, *IQR* Interquartile range, *ULN* Upper limit of normal, *CBD* common bile duct

### CBD stones or sludge on ERCP

In 155 of the 707 patients, no CBD stones or sludge were visualized during ERCP. This resulted in a negative ERCP rate of 22%. In the 485 patients who underwent a strict work-up according to the ASGE 2019 guidelines, the negative ERCP rate was comparable (20%; *n* = 88) (Fig. 1). 81 (12%) patients developed ERCP-related adverse events: post-ERCP pancreatitis (*n* = 54), bleeding (*n* = 21), perforation (*n* = 5), and cholangitis (*n* = 6). The adverse events were evenly distributed between those with a negative [*n* = 21 (14%)] and positive ERCP (*n* = 60 (11%); *P* = 0.36, Table 2). Of the 155 patients with a negative ERCP, 132 patients (85%) still received a sphincterotomy.

**Table 2:** Imaging and complications in patients with negative and positive ERCP

	Negative ERCP *N* = 155	Positive ERCP *N* = 552	*P* value
Abdominal US	136 (88%)	481 (87%)	0.84^a^
Additional imaging (EUS/MRCP/CT)	49/136 (36%)	225/481 (47%)	**0.039**^**a**^
No abdominal US	19 (12%)	71 (13%)	0.84^a^
Additional imaging (EUS/MRCP/CT)	13/19 (68%)	59/71 (83%)	0.40^a^
Abdominal US	136 (88)	481 (87)	0.84^a^
Days before ERCP	2 (1–6)	2 (1–8)	0.68^b^
CT-scan	20 (13%)	70 (13%)	0.94^a^
Days before ERCP	7 (4–27)	5 (2–15)	0.22^b^
EUS	42 (27)	183 (33)	0.15^a^
Days before ERCP	3 (1–7)	1 (0–4)	**0.001**^**b**^
MRCP	14 (9%)	68 (12%)	0.26^a^
Days before ERCP	7 (2–22)	9 (2–28)	0.88^b^
EUS and/or MRCP	54 (35%)	242 (44%)	**0.045**^**a**^
Adverse events of ERCP	21 (14%)	60 (11%)	0.36^a^
Pancreatitis	14 (9%)	40 (7%)	0.46^a^
Bleeding	5 (3%)	16 (3%)	1.0^c^
Perforation	2 (1%)	3 (< 1%)	0.58^c^
Cholangitis	0 (0%)	6 (1%)	0.30^c^

Bold indicates *P* < 0.05 as significant

Data are *n* (%) or median (IQR). ^a^Pearson’s Chi-Square test; ^b^Mann–Whitney *U* test; ^c^Fisher’s Exact test

*IQR* interquartile range, *US* Ultrasound, *EUS* endoscopic ultrasonography, *MRCP* magnetic resonance cholangiopancreatography, *CT* computed tomography, *ERCP* endoscopic retrograde cholangiopancreatography

### Pre-ERCP imaging

Almost all patients (*n* = 689 (98%)) underwent imaging leading to ERCP: abdominal US in 617 (87%), CT in 90 (13%), EUS in 225 (32%), MRCP in 82 (11%) patients (Table 1). The majority (*n* = 658 (93%)) had imaging of the biliary system in the 31 days prior to the ERCP (see Appendix Table S3 and S4, which demonstrates the outcome of the additional imaging performed 0–31 days before ERCP and its effect on ERCP outcome). Of all patients who did not receive abdominal US (*n* = 90), 80% (72 cases) received other imaging types (CT/MRCP/EUS). Patients with positive ERCP findings had undergone more additional imaging (EUS/MRCP) before ERCP compared to patients with negative ERCP findings (44% vs. 35%, respectively; *P* = 0.045) (Table 2). The ERCP in patients stratified to the intermediate likelihood group was more often positive when EUS/MRCP was performed (82% vs 69%; *P* = 0.008). The negative predictive value for EUS and MRCP was 22 and 30%, respectively. Positive predictive value for EUS and MRCP was 81 and 85%, respectively. See Appendix Figure S1 and Table S2, which demonstrates the effect of prior additional imaging for ASGE and ESGE.

### Timing of pre-ERCP imaging

**Fig. 2:**
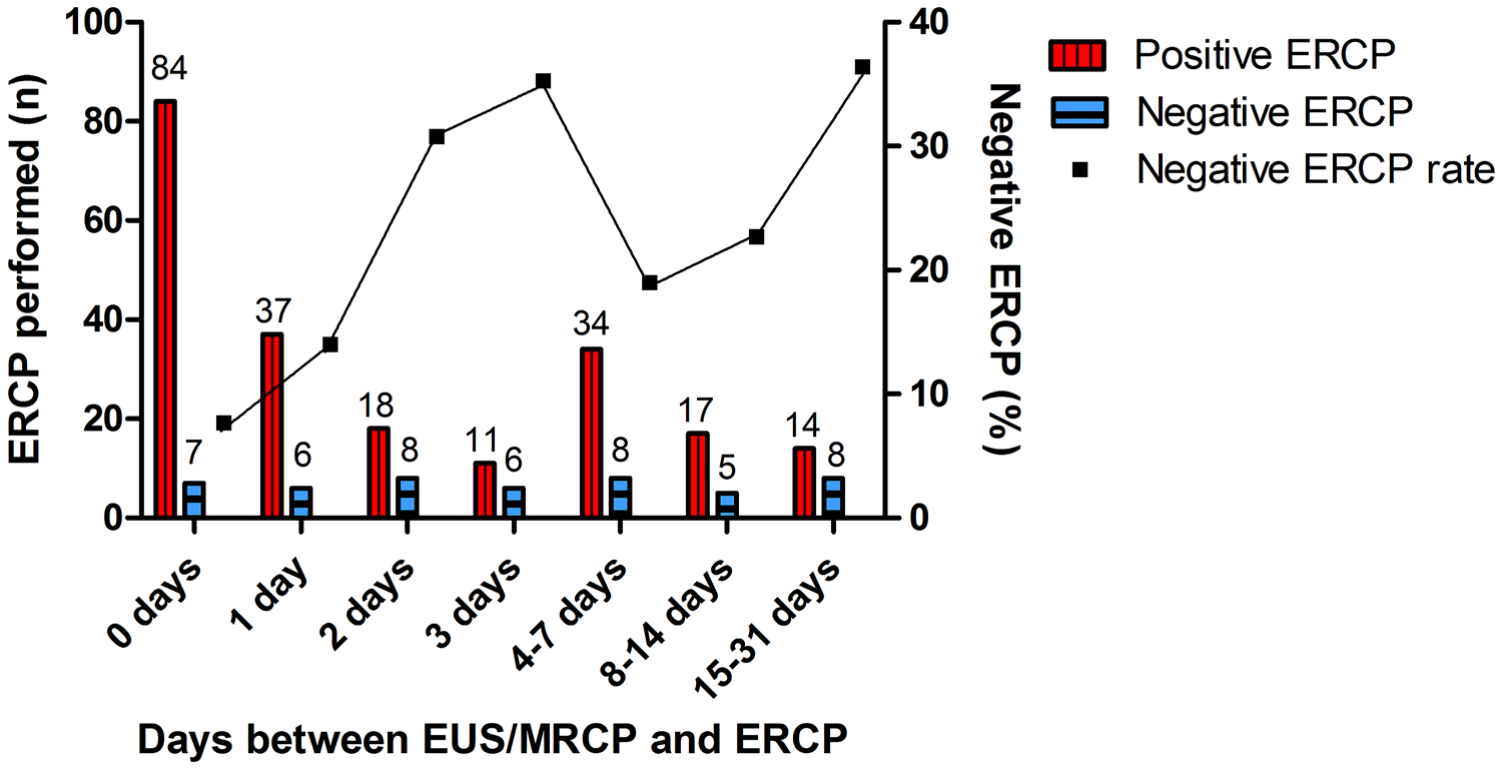
ERCP outcome in patients who underwent pre-ERCP imaging. *EUS* endoscopic ultrasonography, *MRCP* magnetic resonance cholangiopancreatography, *ERCP* endoscopic retrograde cholangiopancreatography

EUS was performed in a significantly shorter time interval before the ERCP compared to the MRCP (median of 1 vs. 8.5 days; *P* < 0.001). The median time interval between EUS and ERCP was longer in those with a negative ERCP for CBD stones (3 days) compared to patients with a positive ERCP (1 day) (*P* = 0.001). Also, the proportion of CBD stone negative ERCPs increases when the time interval between EUS/MRCP and the ERCP became longer (Fig. 2). By performing pre-ERCP imaging by EUS/MRCP within 2 days more CBD stones were visualized during ERCP (87%; *n* = 139/160) compared with more distant imaging (74%; *n* = 76/103) (3–31 days) [odds ratio 2.35; 95% CI 1.25–4.44; *P* = 0.008, number needed to harm (NNH) 7.7].

## Discussion

In 22% of the 707 patients who underwent an ERCP for suspected CBD stones in our prospective multicenter cohort, no CBD sludge or stones was present. When additional pre-ERCP imaging was performed (EUS/MRCP), the chance of CBD sludge or stones during ERCP increased. Timing is of importance: a delay of more than two days between diagnostic imaging (EUS/ MRCP) and ERCP reduces the chance of a positive ERCP. We advocate that additional imaging by EUS or MRCP should be repeated if the time interval exceeds 2 days.

In this study, we demonstrated that EUS or MRCP prior to ERCP increases the proportion of positive ERCPs (*P* = 0.045), which is in line with previous studies [18–21]. We did not find this association for the individual imaging modalities. For MRCPs, this might be as result of the low number of performed MRCPs (82 cases, 12%) and the long time interval between MRCP and ERCP (median of 7 days (positive) and 9 days (negative)). There might be room for improvement since approximately one third (36%) of the patients categorized to the intermediate likelihood group according to ASGE 2019 did not receive an EUS or MRCP. Hence, we would like to emphasize the importance of additional imaging in patients categorized in the intermediate likelihood group for CBD stones when implementing ASGE and ESGE 2019 guidelines (see Appendix Table S2).

In addition, timing of pre-ERCP imaging plays an important role with regard to the probability of CBD stone/sludge at ERCP. A shorter interval between EUS and ERCP (median of 1 vs. 3 days) and immediate prior imaging by EUS or MRCP (≤ 2 days vs. 3–31 days) resulted in more positive ERCPs. However this last finding was mainly due to the contribution of the EUS procedure within 2 days. This time interval should be short because obstructing CBD stones may migrate spontaneously to the duodenum over time [22, 23]. Size of the stone might also be a factor of influence to the probability of spontaneous migration of CBD stones. However, in the case of MRCP/EUS-proven CBD stones, waiting for spontaneously stone migration might not be the best option. Importantly, when ERCP or cholecystectomy is postponed, patients are at risk for gallstone-related complications [24, 25]. In our cohort, 2/29 patients had a CBD stone on abdominal imaging in 1–3 months before ERCP and developed a cholangitis in the time before ERCP (see Appendix Table S5, which demonstrates the outcome of imaging 32–93 days before ERCP). These biliary events could have been prevented by earlier ERCP. Furthermore, it might be beneficial to perform an EUS and ERCP in a single session as opposed to two separate endoscopic procedures with two sedation schemes and two hospital visits. However, the endoscopist has to be trained to perform both EUS and ERCP, which could be a point of attention in the training of future endoscopists. In addition, cost-effectiveness studies show that the costs of routine use of EUS/MRCP and the potential logistical inefficiency of a combined EUS with optional ERCP endoscopy program (in which time has to be reserved for ERCPs that may not be necessary after negative EUS) could be compensated by the reduction in health care expenditure because of avoidance of (unnecessary) ERCPs and ERCP-related complications [26–30].

Our study found that a post-ERCP pancreatitis occurred in 14 (9%) of the 155 patients with a negative ERCP. Better selection would have avoided these complications. More accurate patient selection can be achieved by performing pre-ERCP EUS or MRCP in case of suspected CBD stones irrespective of guideline stratification. The associated NNH for performing pre-ERCP EUS/MRCP to reduce the incidence of post-ERCP pancreatitis from 9 to 0% is 11.1 patients. To prevent all ERCP-related complications in this group (14%) the NNH is even lower (7.1 patients). There is limited data available on the rate of negative ERCPs considered acceptable to ERCPists. A survey among gastroenterologist performed in 2012 determined that a negative ERCP rate of 25% was considered acceptable [31]. Since that time there is a more wide-spread availability of EUS (and MRCP) and, therefore, we believe 25% negative ERCP rate is not acceptable these days [32].

A major aim of the revised ASGE guideline was to reduce CBD stone negative ERCPs by enlarging the intermediate likelihood group through excluding patients from the former high likelihood group [5]. In our cohort, we observed similar rates of stratification to the high likelihood groups (62 and 56% in the ASGE 2010 and 2019, respectively), compared to other studies who did not [13, 14]. A reason might be that we stratified patients later in their disease course and, therefore, they reach the higher threshold for the bilirubin levels as established in 2019 criteria [4, 5]. Nevertheless, our proportion of negative ERCPs for high likelihood patients (21%) is in line with previous studies (17–34%) [10, 13, 14, 33, 34]. It is possible that the relatively large proportion of our cohort (42%, 296 cases) who underwent pre-ERCP EUS/MRCP imaging accounts for the observation that our overall negative ERCP rate of 22% is relatively low compared to previous studies with overall rates between 11 and 62% [10–14, 33, 35].

For any diagnostic study to potentially impact future clinical practice, results should be obtained in a representative population and setting. This multicenter study closely mimics daily practice and contains a large number of patients of both university and large teaching hospitals. We believe that our results are generalizable to daily practice and are relevant to clinicians managing patients with suspected CBD stones.

We acknowledge several potential limitations of this analysis. First, in the original multicenter randomized trial, patients who underwent pre-ERCP imaging and had no presumptive evidence of sludge or stones in the CBD, did not proceed to ERCP were not included in the current study. This might have introduced selection bias in the patients stratified, especially to the intermediate likelihood group. Second, there was no uniform timing for risk classification, because the study protocol did not include a recommended work-up for a patient. Standardized work-up was not feasible due to the diversity within the population of in- and outpatient performed ERCP and the related decision-making toward ERCP. This may have influenced the distribution of patients among the risk categories, but reflects the heterogeneity of the general population in which ERCP is considered. Ideally, a prospective cohort of all patients with suspected sludge or stones in the CBD, referred for ERCP, needs to be considered, in which all patients should undergo work-up according to the guideline.

In conclusion, even in the current era CBD sludge or stones are absent in one out of five ERCPs performed for suspected CBD stones despite previous imaging or work-up according to ASGE 2019 guideline. EUS or MRCP before ERCP should be considered in all patients to allow optimal patient selection and avoiding potential overutilization of diagnostic ERCP. When time interval between EUS/MRCP exceeds 2 days imaging should be repeated to increase the yield of ERCP for suspected CBD stones.

## Supplementary Information

Below is the link to the electronic supplementary material.

Supplementary file1 (DOCX 276 KB)

## Abbreviations

ASGE: American society for gastrointestinal endoscopy
CBD: Common bile duct
CI: Confidence interval
CT: Computed tomography
ERCP: Endoscopic retrograde cholangiopancreatography
EUS: Endoscopic ultrasound
IQR: Interquartile range
MRCP: Magnetic resonance cholangiopancreatography
NNH: Number needed to harm
NPV: Negative predictive value
PPV: Positive predictive value
SD: Standard deviation
ULN: Upper limit of normal
US: Ultrasound
LR: Likelihood ratio
