# Supplementary material for: Suspected common bile duct stones: reduction of unnecessary ERCP by pre-procedural imaging and timing of ERCP
Source: Surg Endosc. 2022 Sep 26;37(2):1194–202. doi: 10.1007/s00464-022-09615-x (PMC9944135; doi:10.1007/s00464-022-09615-x)
Supplement: Supplementary file 1 — Supplementary file1 (DOCX 276 KB) [file 464_2022_9615_MOESM1_ESM.docx]

**Supplementary Information**

This appendix has been provided by the authors to give readers additional information about their work.

**Suspected common bile duct stones: reduction of unnecessary ERCP by pre-procedural imaging and timing of ERCP**

Christina J. Sperna Weiland, Evelien C. Verschoor, Alexander C. Poen, Xavier J.M.N. Smeets, Niels G. Venneman, Abha Bhalla, prof. Ben J.M. Witteman, Hester C. Timmerhuis, Devica S. Umans, prof. Jeanin E. van Hooft, prof. Marco J. Bruno, prof. P. Fockens, Robert C. Verdonk, prof. Joost P.H. Drenth, Erwin J.M. van Geenen for the Dutch Pancreatitis Study Group

**Contents**

[Table S1. Demonstrating details of ASGE 2010 and 2019 and ESGE 2019 guideline 3](#_Toc72139864)

Figure S1. Patient selection and risk stratification of included patients with suspected common bile duct stones 4

Table S2. Demonstrating prediction and adherence to the recommendations of American and European guidelines for management of patients with suspected choledocholithiasis in relation to the presence of choledocholithiasis. 5

Table S3. Demonstrating imaging 0-31 days before ERCP 6

Table S4. Demonstrating imaging day 0-31 before ERCP in patients with negative and positive ERCP 6

[Table S5. Demonstrating the outcome of imaging in patients that received imaging 32-93 days before ERCP and ERCP indication for biliary event in these patients (n=72)](#_Toc72139868) 7

| **Table S1. Demonstrating details of ASGE 2010 and 2019 and ESGE 2019 guideline on the management of common bile duct stones** | | |
| --- | --- | --- |
| **ASGE 2010^4^** | **ASGE 2019^5^** | **ESGE 2019^6^** |
| Very strong  *CBD stone on transabdominal US*  *Clinical ascending cholangitis^#^*  *Bilirubin >4 mg/dL*  Strong  *Dilated CBD on abdominal US*^◊^  *Bilirubin level 1.8-4 mg/dL*  Moderate  *Abnormal liver biochemical test* other than bilirubin*  *Age >55 years*  *Clinical gallstone pancreatitis*  Assigning a likelihood of choledocholithiasis  based on clinical predictors  High: Presence of any very strong predictor or Presence of both strong predictors  Low: No predictors present  Intermediate: All other patients | High likelihood of choledocholithiasis (1 out of 3)  *Common bile duct stone on US/cross-sectional imaging*  *Clinical ascending cholangitis*  *Total bilirubin >4 mg/dL and dilated common bile*  *duct on abdominal US/cross-sectional imaging*^◊^  Intermediate likelihood of choledocholithiasis (1 out of 3)  *Abnormal liver biochemical tests*  *Age >55 years*  *Dilated common bile duct on US/cross-sectional imaging*  Low likelihood of choledocholithiasis: No predictors present | High likelihood of choledocholithiasis (1 out of 2)  *Common bile duct stone on US*  *Clinical ascending cholangitis*  Intermediate likelihood of choledocholithiasis (1 out of 2)  *Abnormal liver function tests*  *Dilated common bile duct on abdominal US*^◊^  Low likelihood of choledocholithiasis: No predictors present |
| # according to Tokyo Guidelines (TG) 2018  * At least one liver test value was above the upper limit of normal  ◊ more than 6mm in patients without a history of cholecystectomy and more than 8mm with a prior cholecystectomy | | |

**Figure. S1: Patient selection and risk stratification of included patients with suspected common bile duct stones**


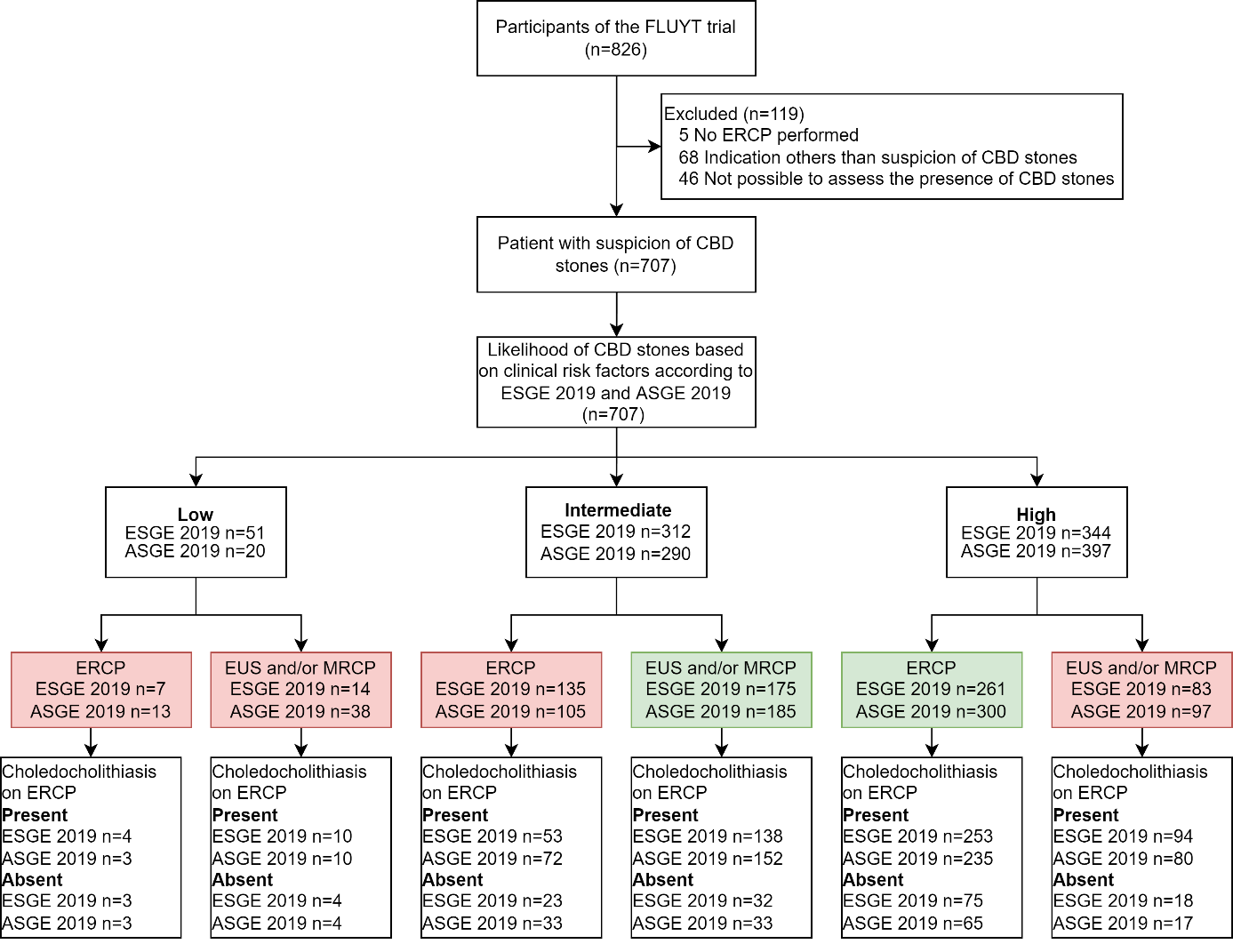


ASGE=American Society for Gastrointestinal Endoscopy, CBD=common bile duct, ESGE=European Society for Gastrointestinal Endoscopy ERCP=endoscopic retrograde cholangiopancreatography EUS=endoscopic ultrasonography, MRCP=magnetic resonance cholangiopancreatography.

Red box=work-up according to real-world practice; Green box=work-up according to ESGE or ASGE 2019 guideline.

| **Table S2. Demonstrating prediction and adherence to the recommendations of American and European guidelines for management of patients with suspected choledocholithiasis in relation to the presence of choledocholithiasis** | | | |
| --- | --- | --- | --- |
|  | **EUS and/or MRCP prior to ERCP** | **Proceeded directly to ERCP** | **P value** |
| **ASGE 2010** |  |  |  |
| High risk  Negative ERCP  Positive ERCP | 112/440 (26%)  18 (16%)  94 (84%) | 328/440 (75%)  75 (23%)  253 (77%) | 0.128^a^ |
| Intermediate risk  Negative ERCP  Positive ERCP | 170/246 (69%)  32 (19%)  138 (81%) | 76/246 (31%)  23/76 (30%)  53/76 (70%) | **<0.001^a^** |
| Low risk  Negative ERCP  Positive ERCP | 14/21 (68%)  4 (29%)  10 (71%) | 7/21 (33%)  3 (43%)  4 (57%) | 0.638^b^ |
| **ASGE 2019** |  |  |  |
| High risk  Negative ERCP  Positive ERCP | 97/397 (24%)  17 (18%)  80 (82%) | 300/397 (76%)  65 (22%)  235 (78%) | 0.381^a^ |
| Intermediate risk  Negative ERCP  Positive ERCP | 185/290 (64%)  33 (18%)  152 (82%) | 105/290 (36%)  33 (31%)  72 (69%) | **0.008^a^** |
| Low risk  Negative ERCP  Positive ERCP | 14 (70%)  4 (29%)  10 (71%) | 6/20 (30.0%)  3 (50.0%)  3 (50.0%) | 0.613^b^ |
| **ESGE 2019** |  |  |  |
| High risk  Negative ERCP  Positive ERCP | 83/344 (24%)  14 (17%)  69 (83%) | 261/344 (76%)  55 (21%)  206 (78%) | 0.405^a^ |
| Intermediate risk  Negative ERCP  Positive ERCP | 175/312 (56%)  32 (18%)  143 (82%) | 137/312 (44%)  43 (31%)  94 (69%) | **0.007^a^** |
| Low risk  Negative ERCP  Positive ERCP | 38/51 (75%)  8 (21%)  30 (79%) | 13/51 (25%)  3 (23%)  10 (77%) | 1.000^b^ |
| ^a^ Pearson’s Chi-Square test; ^b^ Fisher Exact test  ASGE=American Society for Gastrointestinal Endoscopy, ESGE=European Society of Gastrointestinal Endoscopy, ERCP=endoscopic retrograde cholangiopancreatography | | | |

| **Table S3. Demonstrating imaging 0-31 days before ERCP** | |
| --- | --- |
|  | **N** |
| Abdominal US  CBD stone or sludge*****  Dilated CBD*****  Previous cholecystectomy  No previous cholecystectomy | 568  185 (34%)  364 (66%)  75 (21%)  289 (79%) |
| Imaging positive for choledocholithiasis prior to ERCP  MRCP  EUS  CT-scan | 56/65 (86%)  212/220 (96%)  38/77 (49%) |
| Data are n (%). IQR=Interquartile range, ULN=upper limit of normal, US=Ultrasound, EUS=endoscopic ultrasonography, MRCP=magnetic resonance cholangiopancreatography, CT=computed tomography, ERCP=endoscopic retrograde cholangiopancreatography, CBD=common bile duct.  *****18 missing values | |

| **Table S4 Demonstrating imaging day 0-31 before ERCP in patients with negative and positive ERCP** | | | |
| --- | --- | --- | --- |
|  | **Negative ERCP**  **N = 155** | **Positive ERCP**  **N = 552** | **P value** |
| Abdominal US  Days before ERCP  Additional imaging (EUS/MRCP/CT)  No abdominal US  Additional imaging (EUS/MRCP/CT) | 127 (82%)  2 (1-5)  43/127 (34%)  28 (18%)  17/28 (61%) | 441 (80%)  2 (1-5)  189/440 (43%)  111 (20%)  74/111 (67%) | 0.57^a^  0.42^b^  0.13^a^  0.57^a^  0.42^a^ |
| EUS  Days before ERCP | 41 (27%)  3 (1-7) | 179 (32%)  1 (0-3%) | 0.16^a^  **0.000^b^** |
| MRCP  Days before ERCP | 13 (8%)  7 (2.5-17.5) | 52 (9%)  5 (1-14) | 0.69^a^  0.31^b^ |
| EUS and/or MRCP | 52 (34%) | 226 (41%) | 0.096^a^ |
| CT-scan  Days before ERCP | 17 (11%)  7 (3.5-11.5) | 60 (11%)  4.5 (2-10.8) | 0.97^a^  0.15^b^ |
| Data are median (IQR) or n (%). US=Ultrasound, EUS=endoscopic ultrasonography, MRCP=magnetic resonance cholangiopancreatography, CT=computed tomography, ERCP=endoscopic retrograde cholangiopancreatography. ^a^ Pearson’s Chi-Square test; ^b^ Mann-Whitney U test; ^c^ Fisher Exact test. | | | |

| **Table S5. Demonstrating the outcome of imaging in patients that received imaging 32-93 days before ERCP and ERCP indication for biliary event in these patients (n=72)** | | | | |
| --- | --- | --- | --- | --- |
|  | **Abdominal US (n=49)** | **MRCP**  **(n=17)** | **EUS**  **(n=5)** | **CT-scan**  **(n=13)** |
| CBD stone or sludge | 1 | 6 | 1 | 1 |
| CBD dilatation | 17 | 1 | 0 | 6 |
| CBD stone or sludge + CBD dilatation | 6 | 9 | 3 | 2 |
| No abnormalities seen | 21 | 0 | 1 | 4 |
| No result | 4 | 1 | 0 | 0 |
|  |  |  |  |  |
| - Patients with a biliary complication (cholangitis) due to ERCP delay after imaging positive for CBD stone/sludge (n=29) | 1 | 1 | 0 | 0 |
| US=Ultrasound, EUS=endoscopic ultrasonography, MRCP=magnetic resonance cholangiopancreatography, CT=computed tomography, ERCP=endoscopic retrograde cholangiopancreatography, CBD=common bile duct. | | | | |
